# Variation of Binge Eating One Year after Roux-en-Y Gastric Bypass and Its Relationship with Excess Weight Loss

**DOI:** 10.1371/journal.pone.0167577

**Published:** 2016-12-19

**Authors:** Luciano Billodre Luiz, César Luis de Souza Brito, Letícia Manoel Debon, Lívia Nora Brandalise, Juliana Tainski de Azevedo, Karin Daniele Monbach, Luísa Schmidt Heberle, Cláudio Corá Mottin

**Affiliations:** 1 Center of Obesity and Metabolic Syndrome, São Lucas Hospital of Pontifical Catholic University of Rio Grande do Sul, Porto Alegre, Brazil; 2 Faculty of Medicine, Pontifical Catholic University of Rio Grande do Sul, Porto Alegre, Brazil; National Institue on Drug Abuse, UNITED STATES

## Abstract

**Background:**

Bariatric surgery is the most effective treatment for obesity classes II and III. However, some patients do not get the desired results or initially lose and then regain the lost weight. Identifying these individuals early on and treating them adequately remains a challenge. As binge eating directly affects food intake, the study of this symptom and its relation to bariatric surgery and its results is increasing, because it appears to have an influence on the results of surgery.

**Objectives:**

This study aimed to see how binge eating changes, measured with the Binge Eating Scale, interferes in the % excess weight loss one year after Roux-en-Y gastric bypass.

**Methods:**

We conducted a cross-sectional study with 149 patients older than 18 years who were evaluated one year after undergoing Roux-en-Y gastric bypass. The variation in the intensity of binge eating was measured with the pre- and postoperative Binge Eating Scale scores.

**Results:**

The variation of one unit in the Binge Eating Scale implied an inverse variation of 0.41% of % excess weight loss (p<0.05). The correlation coefficient between the variation of binge eating and the % excess weight loss was -0.186 (p = 0.033). The correlation coefficient between the binge eating symptoms one year after surgery and the % excess weight loss was -0.353 (p<0.001).

**Conclusions:**

There was a correlation between the variation of binge eating one year after gastric bypass and the % excess weight loss. The correlation between binge eating and the % excess weight loss was greater after the surgery than it was at the preoperative stage. This study provides new, valuable information on the intensity and variation of binge eating symptoms one year after gastric bypass, which, to the best of our knowledge, have not been studied in depth earlier.

## Introduction

Bariatric surgery is the most effective treatment for the control of classes II and III obesity and reduces mortality and controls related chronic diseases [1]. However, some patients either do not lose, or lose and regain, part of the lost weight, and identifying the reasons for this is still a major challenge [2]. This population, in comparison to individuals of normal weight, shows a higher prevalence of psychiatric disorders, including binge eating disorder (BED) [3–6]. As eating behavior directly affects any treatment for obesity, the study of this behavior and its alterations has been increasing in recent years, since it may be related to the response to the surgery [6,7].

New technologies such as functional magnetic resonance imaging have identified the brain circuits related to eating behavior, mainly through the visual stimulus of palatable food, leading to the activation of these circuits [8]. Brain circuitry related to reward and attention, which had been highly active preoperatively, diminished patients’ response to the same stimulus shortly after bariatric surgery [9–11]. This is the reverse of what happens in diets where there is significant caloric deprivation; the activation of these circuits increases. This also increases the chances that these individuals will gain weight in the future [12,13].

Attempts to verify whether the presence of BED is a weight loss predictor have produced controversial results [6]. It is known that after surgery, BED tends to interfere negatively with reduction of the excess weight [14]. Patients with binge eating (BE) symptoms were found to have lost less weight and have worse outcomes than patients without BE symptoms at two years after surgery [15]. As noted by Gavin Meany et al., it is difficult to compare the results in the literature because many methods are used to diagnose BE; these different methods produce discrepancies in the results [6]. Evaluating the intensity of the symptoms from the lightest to the most severe, without setting a cut-off point or a clinical criterion for the presence or absence of BE, is a way to check its influence on the surgical outcome without going into the issue of defining an objective or a clinical criterion. Studying by category whether or not BE exists in the case being examined and determining its relationship with the surgical results runs into the classification difficulties described above, given that BE manifests in the clinic as being on a spectrum from a single, mild symptom to a very intense and varied presentation. In addition, this marker is also less reliable clinically.

However, defining BE remains a major challenge. As the surgery imposes a restriction on food intake, the feeling of loss of food control has been studied as an isolated BE element in this population [16,17]. However, behavioral symptoms such as the amount ingested, the rate of food intake, secretive eating, among others, are also part of the syndrome and cannot be forgotten in the study of BE, even in operated patients [18]. To our knowledge, no study has evaluated the intensity and variation of BE symptoms.

The main objective of our study is to see how the variation in intensity of BE—measured using the Binge Eating Scale (BES)—interferes with the percentage of excess weight loss (%EWL) one year after surgery. In addition, we propose to verify how the intensity of BE before the surgery and one year after the procedure, as well as the presence of BED, relate to the %EWL. We found a correlation between the variations in BE one year after gastric bypass and the %EWL. The correlation between intensity of binge eating and the %EWL was greater after the surgery than it was at the preoperative stage.

## Materials and Methods

### Participants and design

We conducted a cross-sectional study of 149 individuals, all older than 18 years, who were assessed one year after undergoing Roux-en-Y gastric bypass (RYGB) in a university hospital. Demographic and clinical data were collected from the patients’ records, as well as from the preoperative BES. At the one-year follow-up consultation and revision time, BES was re-applied and the patient’s weight was verified. Of the 149 individuals, 17 were excluded from the study: one woman for being pregnant (interference body mass index), 12 for loss of preoperative data (had no BES), and four due to incorrect reporting of BES in the postoperative evaluation. All patients underwent routine pre- and postoperative consultations and treatments. All participants agreed to participate in the study and freely signed informed consent. The São Lucas Hospital Ethics Committee at PUCRS (Pontifícia Universidade Católica do Rio Grande do Sul) approved the study.

### Measurement of the intensity of binge eating

The BES is a self-administered instrument developed by Gormally et al. to measure the severity of BE [18] and was translated and adapted in Portuguese in 2001 [19]. It comprises a Likert scale and a list of 16 items with 62 affirmatives; individuals select the answer that best represents their response. The final score is the sum of the points of each item and ranges from 0 to 46. The BES is a tested and reliable instrument for use with candidates for bariatric surgery both before and after the procedure [20]. In addition to quantifying the intensity of BE, the BES has been shown to be an effective instrument for screening patients with BED. BES values above 17 suggest, with a sensitivity of 94% and a specificity of 76%, diagnostic of BED in candidates for bariatric surgery [20].

The BES quantifies BE in its entirety. It contemplates two different factors of compulsion: feelings/cognition and behavioral manifestations. Even if there is a change in the presentation of BE after surgery (for example, more feeling or cognitive manifestations and fewer behavior manifestations) the scale can detect and score these modifications. As previous studies in bariatric populations have shown, assessing the BES separately in each of its factors [21] did not alter the results; neither gain nor any difference was found.

### Variation of the intensity of binge eating

To study the variation of the symptoms of BE, we measured the difference of the BES score between the pre- and postoperative stages of the treatment. We called the result Delta BES (Delta BES = postoperative BES score—preoperative BES score).

### Statistical analysis

The data were typed into an Excel spreadsheet and then exported to the SPSS Inc., PASW Statistics for Windows, Version 18.0. (Chicago, IL, USA) for statistical analysis. The categorical variables were described as frequency and percentage. Quantitative variables with symmetric distribution were described using the average and the standard deviation. The categorical variables were compared using the Chi-square test or Fisher's exact test. The quantitative variables with symmetric distribution were compared between two categories with Student’s t test for independent samples. Variables with asymmetric distribution were compared using the Wilcoxon test (for times) and the Mann-Whitney test (for groups). To evaluate the correlation between quantitative variables, we used Spearman’s correlation coefficient. A significance level of 0.05 was considered for the established comparisons.

## Results

### Characteristics of the sample

Our sample consisted of 132 individuals, with an average age of 38.27 years (standard deviation 10.07), with 105 (79.5%) women. Their pre- and postoperative characteristics are presented in Table 1.

**Table 1:** Characteristics of the patients in our study.

	Preoperative	One year after surgery
**Height**	1.64 m (0.094m)	-
**Weight**	131.16 Kg (28.29Kg)	86.08 Kg (19.15Kg)
**BMI**	48.31 Kg/m^2^ (7.92 Kg/m^2^)	31.74 Kg/m^2^ (5.70 Kg/m^2^)
**BES Score**	13.58 (7.21)	6.64 (6.44)
**Excess weight**	63.43 Kg (24.06Kg)	-
**%EWL**	-	73.99% (16.57%)
**Delta BES**	-	-6.94 (7.27)

Data are presented as means and standard deviations. BMI, body mass index; BES, binge eating scale; %EWL, % of excess weight loss; Delta BES, BES at one year—preoperative BES.

### BE and %EWL

We found a significant relationship between the change in intensity of BE, measured using the Delta BES one year after surgery, and %EWL. The variation of one unit in the BES implied an inverse variation of 0.41% EWL (p<0.05) (95% confidence interval [CI] -0.79 to -0.02).

**Fig 1:**
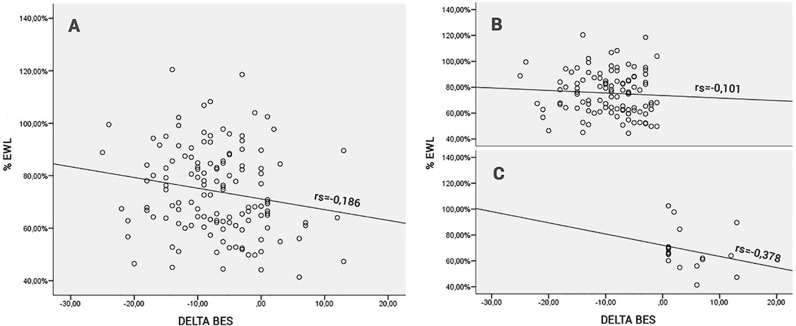
Correlation between the variation of BE (Delta BES) and %EWL. A Correlation coefficient between %EWL and Delta BES and the distribution of the entire sample. rs = -0.186 p = 0.033B Correlation coefficient and distribution of patients who had a negative Delta BES. rs = -0.101 p = 0.306C Correlation coefficient and distribution of patients who had a positive Delta BES. rs = -0.378 p = 0.122
%EWL, % of excess weight loss; Delta BES, BES at one year—preoperative BES; rs, correlation coefficient.

Fig 1 shows the distribution of patients according to the variation of the intensity of the BE, the %EWL, and the correlation coefficient among these variables.

One hundred five (79.54%) subjects had BE symptoms that decreased; i.e., they had a negative Delta BES and an average %EWL of 75.46% (16.53%SD). The 18 (13.63%) subjects who had increased intensity BE or a positive Delta BES had an average %EWL of 68.25 (16.18% SD). Ten individuals maintained the same BES score.

**Fig 2:**
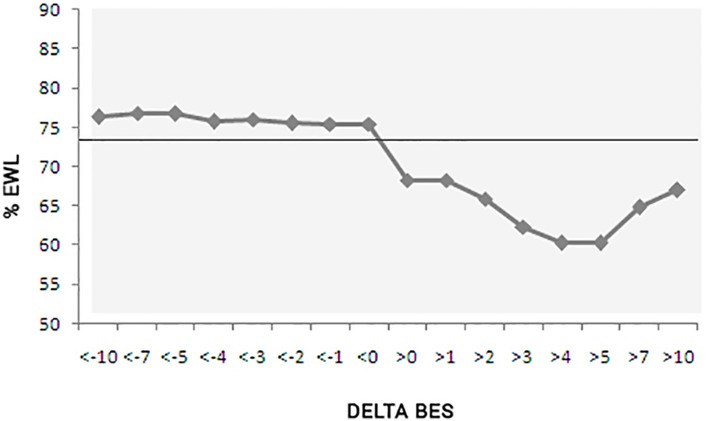
%EWL according to the variation of BE (Delta BES), progressively—greater and lower than 0. %EWL, % of excess weight loss; Delta BES, BES at one year—preoperative BES; General average of %EWL = 73.99%.

Fig 2 shows the overall average %EWL of the study population and the subgroup averages of progressively varying the intensity of BE larger than 1 and smaller than -1.

**Fig 3:**
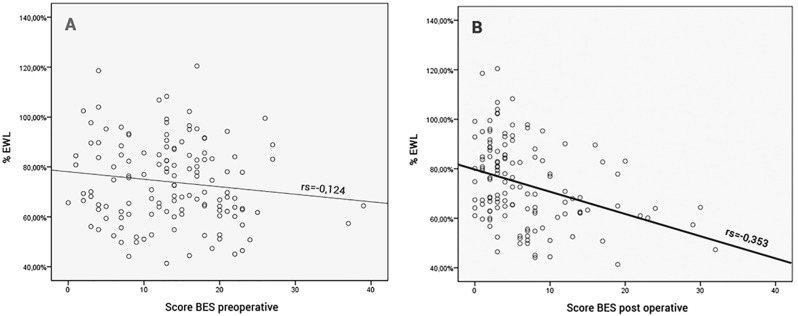
Correlation between BES and %EWL in the preoperative stage and one year after surgery. A BES score in the preoperative stage and its correlation with %EWL. rs = -0.124 p = 0.156B BES score one year after surgery and its correlation with %EWL. rs = -0.353 p<0.001
%EWL, % of excess weight loss; Delta BES, BES at one year - preoperative BES; rs = correlation coefficient.

Fig 3 shows the intensity distribution of the BE symptoms before and one year after surgery and the correlation coefficients with %EWL.

### BED and %EWL

The 39 (29.54%) patients who presented with BES scores greater than 17 (probable diagnosis of BED) before surgery had a %EWL average of 68.52% of versus a %EWL average of 76.28% in those who did not have the disorder. Individuals with BED before surgery lost an average of 7.76% less excess weight after one year compared with those with no diagnosis (p<0.01) (95% CI 13.5% to 2.02%). Of the individuals with BED, 82.1% did not have this diagnosis after surgery (p<0.05) (95% CI 66.5% to 92.5%).

The 10 (7.58%) individuals with a BES score greater than 17 (probable diagnosis of BED) one year after surgery had an average %EWL of 62.15% against a %EWL average of 74.96% for those who did not have the disorder. Individuals with BED one year after surgery lost an average of least 12.81% excess weight than those without the diagnosis (p<0.01) (95% CI -21.96 to -3.65).

Of the 10 individuals who presented with a diagnosis of BED after one year, only three did not have this diagnosis previously.

## Discussion

There is a correlation between the variation of the intensity of BE symptoms one year after RYGB and the %EWL. This is a new finding, as previous studies have evaluated how the presence of BE interferes with weight loss, but did not take into account its intensity or its variation [6,22–24]. A variation of one point in the BES score one year after the procedure results in a difference of 0.41%EWL.

As seen in Fig 2, the difference in %EWL between the groups with improved BE symptoms and those whose BE symptoms worsened one year after surgery is evident. Groups of patients with worsened BE symptoms lost less %EWL, even when the variation of the BES score was as small as 1 or 2 points. It was also possible to observe a certain uniformity in the %EWL in the groups that had a decrease in the intensity of BE symptoms, even when the variation increased. This is different from that observed in the groups with worsening BE symptoms, who had a tendency to show lower loss of %EWL as the variation increased until the group with a Delta BES > 7 was reached. The general correlation coefficient of Delta BES with %EWL was -0.186, but when we evaluated the group with a positive Delta BES separately, we found that this correlation increased to -0.378. However, due to the small number of patients demonstrating an increase in BE, this value was not statistically significant at p = 0.122. Although these data showed that the worsening of BE symptoms correlated more strongly with the %EWL, further studies with a larger sample are necessary to confirm this finding.

The intensity of BE one year after surgery showed a significant correlation with the %EWL (-3.53 correlation coefficient). This finding reinforces the conclusions of previous studies indicating that poorer surgical results will be obtained when BE is present after the surgery [17,24,25]. Furthermore, we found a correlation between the intensity of BE and %EWL, different from the studies cited above that correlate the presence of BE, BED, and the loss of control of eating but do not consider its intensity. That is, the higher the intensity of BE is one year after surgery, the lower the %EWL will be. We found no correlation between the symptoms of BE before surgery and %EWL. This finding adds to a series of controversial results on the subject, shown by Gavin Meany et al. in their review on the subject [6].

BED showed a prevalence of 29.54% before surgery and 7.58% after one year. These data agree with the averages found in other studies that range between 14% and 55.5% before the procedure and 3% and 37.5% after surgery [6]. Individuals with BED lost less weight than those without the diagnosis both before and after surgery. However, this difference was more marked when the diagnosis was present after the procedure.

Our work has the limitations of being uni-centered—the population consists of mostly of Caucasian women, making it difficult to extrapolate the results to populations with different characteristics. Evaluation of improvement or worsening of symptoms was not correlated with the clinical treatment received by each individual. The diagnosis of BED accomplished with BES tends to be overestimated because, although it is a very sensitive measurement, it is not very specific [20]. Sallet et al. showed that after two years of surgery, the relationship between BE and the surgical results can be seen more clearly [23]. Even though our work indicates a trend, longer follow-up is needed to elucidate more clearly how the variation of the symptoms of BE interfere with the %EWL.

## Conclusion

Summarizing, the variation of BE intensity one year after RYGB interferes with the %EWL. The increase of the intensity of the BE reduces the %EWL independently of the patients’ preoperative levels. There is also a correlation between the intensity of BE one year after surgery and the %EWL. The stand-alone intensity of the BE in the preoperative stage is not a relevant parameter for the %EWL after one year. The presence of a BED diagnosis interferes negatively with the %EWL, both in the preoperative stage and one year after the surgery, but is more intense when present after the procedure. This study provides new, valuable information on the intensity and variation of BE symptoms one year after RYGB, which have not been studied in depth before.

## Supporting Information

S1 Dataset Dataset of research. (XLS)

S1 Checklist STROBE checklist. (DOCX)
